# Clarifying TID and TD in sport: an integrative phase-based framework of talent assessment and promotion

**DOI:** 10.3389/fpsyg.2026.1817616

**Published:** 2026-04-29

**Authors:** Fabienne Döringer, Mark Pfeiffer

**Affiliations:** Department of Theory and Practice of Sports, Institute of Sports Science, Johannes Gutenberg University Mainz, Mainz, Germany

**Keywords:** integrative framework, talent assessment, talent development, talent identification, talent promotion, talent in sport

## Abstract

Research on Talent Identification (TID), and Talent Development (TD) in sport has produced a diverse body of theoretical frameworks, empirical findings, and practice-oriented models addressing different aspects of long-term athlete development. While these contributions have substantially advanced understanding of talent-related processes, they vary in their conceptual focus, level of analysis, and use of phase terminology. As a result, empirical findings are often difficult to compare systematically and to situate clearly within the overall process. The present paper proposes an integrative, phase-based framework for Talent Identification and Development that aims to support clearer conceptual alignment between assessment and promotion across the pathway from talent detection to talent verification. Drawing on insights from sport science, developmental psychology, and established diagnostic and developmental models, the framework distinguishes five interconnected phases: talent detection, talent orientation, talent identification, talent selection, and talent verification. Across all phases, assessment and promotion are conceptualized as parallel and continuously interacting dimensions rather than as sequential steps. A central feature of the framework is its explicit consideration of the phase-specific meaning of talent and the changing relevance of assessment-related characteristics and promotion priorities over time. The framework emphasizes that predictive validity is inherently phase-dependent and provides a structured basis for situating empirical findings within their appropriate developmental context. Rather than offering prescriptive solutions or new predictors of future performance, the framework integrates existing theoretical, empirical, and practice-oriented approaches into a coherent process structure. The proposed framework has implications for both research and practice. For research, it offers a reference system that supports phase-sensitive interpretation of findings and encourages longitudinal, context-aware, and person-centered approaches to studying talent development. For practice, it provides a conceptual tool for reflecting on the alignment between assessment decisions and promotion structures within talent systems. Overall, the framework seeks to contribute to a more coherent, developmentally informed, and integrative understanding of talent assessment and promotion in sport.

## 1 Introduction

Research on Talent Identification (TID) as well as Talent Development (TD) in sport has expanded considerably over recent decades, resulting in a diverse body of empirical studies, theoretical models, and practice-oriented frameworks (Nijenhuis et al., 2024; Taylor et al., 2022). These contributions have advanced understanding of long-term performance development by identifying key influencing factors and by proposing structured descriptions of developmental processes (Baker et al., 2017; Vaeyens et al., 2008). At the same time, the field is characterized by substantial conceptual and terminological diversity, which complicates the comparison of empirical findings and the derivation of coherent theoretical and practical implications.

Particularly in the context of talent assessment and talent promotion, the literature contains inconsistent assumptions regarding what constitutes talent, which characteristics are relevant at different phases of development, and which objectives underlie diagnostic or developmental interventions. As a result, empirical findings are often only partially comparable, theoretical debates may proceed in parallel rather than in dialogue, and practical implications for coaches and practitioners remain fragmented (Till and Baker, 2020). This challenge is further intensified by the fact that identical terms are used to describe different segments of the overall process, while different terms are sometimes applied to similar process phases.

A key source of this ambiguity lies in the fact that the object under consideration in the talent process changes systematically over the course of athletic development. In early phases, the focus is often on general prerequisites, whereas later phases increasingly emphasize sport-specific performance capacity, performance stability, and international competitiveness (Lloyd et al., 2015). At the same time, sport is commonly treated as a single, clearly bounded achievement domain with a pronounced motor component, while the considerable heterogeneity of performance demands across different sports is only rarely addressed explicitly. Consequently, the concept of talent refers to different constellations of characteristics (e.g., motor coordination and curiosity in youth vs. technical mastery and competitive resilience in elite athletes), evaluative criteria, and objectives depending on the developmental phase, without these shifts always being made explicit in scientific discourse (Preckel et al., 2020; Subotnik et al., 2011). Statements regarding TID and TD can therefore only be meaningfully interpreted when they are clearly situated within a specific segment of the developmental process.

Against this background, conceptual and structural clarification becomes a central task. Across disciplines, unresolved differences in meaning and implicit assumptions about processes and objects of inquiry can obscure substantive problems and hinder systematic analysis. Applied to the talent discourse, this suggests that progress depends less on the accumulation of additional isolated findings and more on clarifying the underlying process logic and terminology that structure research and practice.

Within this context, talent in sport is understood as referring to individuals who are still in the process of developing toward their individual peak performance level, who demonstrate above-average sport-related performance relative to peers with comparable developmental and training conditions, and who, given appropriate developmental support, have the potential to reach high performance levels in subsequent phases of development (Baker et al., 2012; Güllich, 2013; Hohmann, 2009; Huijgen et al., 2013). Retrospectively, a talent is someone who has already achieved demonstrable top performance in their sporting career (Hohmann, 2009; Spies et al., 2022). TID as *talent identification* refers to the process of selecting the most promising athletes within a given sport with the aim of further developing them toward elite or world class performance (for a review in racket sports, see Nijenhuis et al., 2024). In this manuscript however talent identification is conceptualized more narrowly as one phase within a broader multi-phase assessment process (see Hohmann, 2009; Pion, 2024; Spies et al., 2022). TD as *talent development* in turn is understood here as talent promotion, a parallel and interdependent process that evolves in continuous interaction with assessment. In the remainder of this article, we therefore refer to *Talent Assessment* and *Talent Promotion* and define them as the central terms.

The aim of the present paper is therefore to propose an integrative, phase-based framework for Talent Assessment and Promotion in sport that systematically links diagnostic, developmental, and practice-oriented perspectives. The framework seeks to clarify key concepts, to structure the phases of the overall process, and to make transparent how the relevance of diagnostic and developmental decisions could change across the developmental pathway. It is intended as a point of convergence that integrates different theoretical perspectives and applied approaches within a shared process-oriented structure. In this sense, “integrative” refers to the systematic alignment of existing viewpoints and to the explicit representation of relationships between established approaches, thereby supporting a more differentiated interpretation of empirical findings and facilitating exchange between research and practice.

Building on this starting point, the following section outlines the rationale for an integrative framework before itself is presented and systematically elaborated along the phases from talent detection to talent verification.

## 2 Rationale for an integrative framework in the talent assessment and promotion process

The scientific literature on talent assessment and promotion in sport comprises a wide range of theoretical frameworks, empirical studies, and practice-oriented models. These contributions address different aspects of long-term athlete development and have substantially advanced understanding of performance pathways across sports. At the same time, they vary considerably in their conceptual focus, temporal scope, and level of analysis. While some approaches emphasize sequential phases, others focus on specific decision points, isolated predictors, or particular developmental phases. As a result, it often remains unclear how individual findings relate to one another and to which part of the overall talent assessment and promotion process they apply (Dunn et al., 2025; Till and Baker, 2020). Alongside sport-specific approaches, there is also a substantial body of research outside sport science that examines talent from a more general, domain-transcending perspective, focusing on common developmental mechanisms across achievement domains such as academics, music, or science (Preckel et al., 2020; Subotnik et al., 2011).

One challenge within this discourse concerns the inconsistent use of phase terminology. Terms such as talent detection, talent orientation, talent identification, talent selection, talent development, or talent confirmation are frequently used in overlapping, synonymous, or ambiguous ways across studies and models (Johnston et al., 2018; Vaeyens et al., 2008). In particular, the term development is used inconsistently. While some conceptualizations treat development as a distinct phase within the process (e.g., Zhao et al., 2024), other approaches conceptualize development as an overarching process dimension that spans all phases of talent progression. In the present article, development is used exclusively to denote the overall process of talent assessment and promotion, whereas the framework itself is structured into distinct phases that capture different functional tasks within this process. This distinction is intended to enhance conceptual clarity by avoiding the simultaneous use of development as both a phase label and a process descriptor. Additionally, *identification* (TID) is presented here as one of several phases on the assessment side. Within this manuscript, assessment and promotion are conceptualized as parallel and continuously interacting perspectives, rather than as separate phases.

Closely related to terminological inconsistency is the question of predictive validity in talent assessment. A substantial body of research has demonstrated that reliable long-term predictions of elite performance are difficult to achieve. Predictive relationships are typically limited to relatively short developmental periods or to specific decision contexts, such as selection moments within organized talent systems (Barth et al., 2022). Longitudinal research and recent reviews further indicate that the relevance and meaning of assessment indicators change systematically over time, depending on developmental level, training history, and contextual constraints (Dunn et al., 2025). Accordingly, at the level of assessment, predictors and indicators can be distinguished that are relevant for the respective level or for the subsequent level (see Preckel et al., 2020). Indicators reflect the level of competence already achieved, whereas predictors are derived from developmental dynamics such as responsiveness to coaching, rates of improvement, and stability of engagement over time. These findings suggest that predictive validity depends on the respective developmental phase and that it should first be considered what is relevant for the given phase or the immediately subsequent phase.

Within this context, the distinction between assessment and promotion represents a key conceptual dimension of the talent assessment and development process. In sport science and developmental research, talent development is not understood as a linear sequence in which assessment precedes promotion. Instead, assessment and promotion are conceptualized as continuously interacting dimensions that operate in parallel throughout the entire process (Hohmann, 2009). Information derived from assessment processes does not merely serve selection decisions; it also informs the design, adaptation, and evaluation of developmental environments and support structures (e.g., the use of psychosocial profiling to tailor individualized training environments, as demonstrated in elite youth soccer academies where motivation and self-regulation scores inform coach-athlete communication strategies and training load adjustments (MacNamara et al., 2010; Toering et al., 2009).

Conversely, promotion affects assessment outcomes by shaping the conditions under which performance is expressed. Additional training, targeted support, or increased competitive exposure can lead to short-term performance improvements that directly influence assessment results, such as competition performance or coach evaluations. In contrast, limited access to high-quality training and competition may constrain performance expression, independent of underlying potential. Empirical research has consistently shown that performance outcomes in sport reflect not only individual characteristics but also training history and contextual conditions (Côté and Fraser-Thomas, 2007; Güllich, 2017; Phillips et al., 2010).

On the assessment side, empirical research has employed a wide range of approaches to capture talent-related characteristics across developmental phases. These include physical and motor assessments of speed, power, endurance, and coordination (Deprez et al., 2015), as well as performance-based and game-related indicators derived from training and competition contexts (Höner and Feichtinger, 2016). In addition, psychological characteristics such as motivation, self-regulation, and coping skills have been examined with regard to their relevance for promotion decisions, sustained participation, and progression within talent pathways (MacNamara et al., 2010; Toering et al., 2009).

More recent work has emphasized multidimensional assessment approaches that combine physical, technical, tactical, and psychological indicators, often within longitudinal designs, to examine their relevance across development (Huijgen et al., 2013; Johnston et al., 2018; Schorer et al., 2017). Collectively, these studies illustrate both the breadth of assessment research in talent assessment and development and the challenges associated with interpreting assessment results across different developmental phases, particularly with respect to long-term predictive claims.

In contrast, practice-oriented models primarily address the promotion and organization of athlete development. Frameworks such as the Long-Term Athlete Development model (Balyi and Hamilton, 2004) and the FTEM framework (Foundation, Talent, Elite, Mastery; Gulbin et al., 2013) structure long-term training pathways, define developmental phases, and offer guidance for organizing coaching, competition, and support systems. While frameworks such as LTAD and FTEM provide structured pathways for athlete development, they typically do not explicitly articulate the diagnostic assumptions underlying their phase definitions. For instance, which physical, technical, or psychological indicators are considered relevant at each phase (Dimundo et al., 2023; Pankhurst et al., 2013; Vaeyens et al., 2008). As a result, the empirical basis for these assumptions often remains implicit or unspecified, limiting the transparency of how assessment (not only in the identification phase) and promotion are conceptually linked within these models. As a consequence, assessment and promotion-related perspectives are often treated as separate strands, despite addressing different aspects of the same long-term process (Pankhurst et al., 2013).

Research grounded in complex systems and ecological dynamics emphasizes that talent pathways emerge from continuous interactions between individual characteristics, task demands, and environmental constraints, rather than from linear cause-effect relationships (Abbott et al., 2005). From this perspective, early performance indicators are best understood as provisional expressions shaped by opportunity structures and developmental environments rather than as stable predictors of long-term success (Baker et al., 2017).

In summary the literature reveals both directly and indirectly several conceptual gaps that necessitate a structured integrative approach upon which a framework is introduced in the following section.

- TID as Talent Identification is often treated in the literature as an isolated phase (besides talent detection, selection, etc., see Pion, 2024), although it represents only one component of the broader assessment process → requiring clearer differentiation.
- Promotion is rarely conceptualized in existing approaches as a process running parallel to and mutually influencing assessment → despite their continuous interdependence (see Hohmann, 2009).
- The lack of systematic positioning of existing research leads to terminological and conceptual ambiguities, complicating comparative analysis.
- Psychological parameters and training adaptations are typically not regarded as equally relevant dimensions alongside motor performance (e.g. Mostaert et al., 2022) → necessitating a broader perspective.
- Existing models are often fragmented or focused on single phases → calling for an integrative structure to represent the overall process.

The framework presented in the following section tries to address these requirements by integrating existing assessment-oriented, promotion-oriented, and practice-based approaches into a clear and structured process intended to support both research and applied decision-making.

## 3 Overarching description of the integrative framework

This section presents the integrative phase-based framework for Talent Assessment and Promotion (TAP) introduced in the previous chapter. Drawing on established theoretical, empirical, and practice-oriented contributions, the framework structures the talent development process along four overarching levels: Aptitude, Competence, Expertise, and Transformational Achievement. These levels consolidate and functionally integrate processes that are often described separately in the literature, such as talent detection, talent orientation, talent identification, talent selection, and talent verification.

Importantly, the talent levels are not conceptualized as fixed age bands or rigid, disjunct phases. Rather, they represent functional segments within a long-term developmental process. Although certain age ranges are more commonly associated with specific levels, progression is primarily determined by sport-specific performance demands and by the typical age of peak performance within a given sport. Consequently, the organization of assessment practices, promotion structures, and decision points needs to be aligned with sport-specific developmental trajectories rather than with chronological age alone (Elferink-Gemser and Visscher, 2012). Transitions between levels are gradual, individual-specific, and characterized by overlap rather than by clear thresholds.

Across all levels, the framework distinguishes assessment and promotion as two parallel and continuously interacting dimensions. Assessment refers to the systematic generation, and interpretation of information used to inform decisions within the TAP process. This includes psychological, sport-motor, sport-specific, and anthropometric characteristics assessed through different methods depending on the developmental level and context. Promotion, in contrast, refers to the organization of training, competition, and support environments. Both dimensions address different aspects of the same process and mutually influence each other, as the characteristics that become observable and measurable depend partly on how promotion-related environments are structured and which learning and performance opportunities are provided.

Within the framework, phase-specific predictors are specified to structure the focus of assessment at each developmental level. These predictors are not understood as long-term determinants of elite performance but as characteristics assumed to be diagnostically relevant for the current level or for the transition to the subsequent one. The framework does not aim to determine the long-term prognostic validity of individual characteristics. Instead, it provides a developmental structure within which existing empirical findings and assessment practices can be interpreted in a phase-sensitive manner.

**Figure 1:**
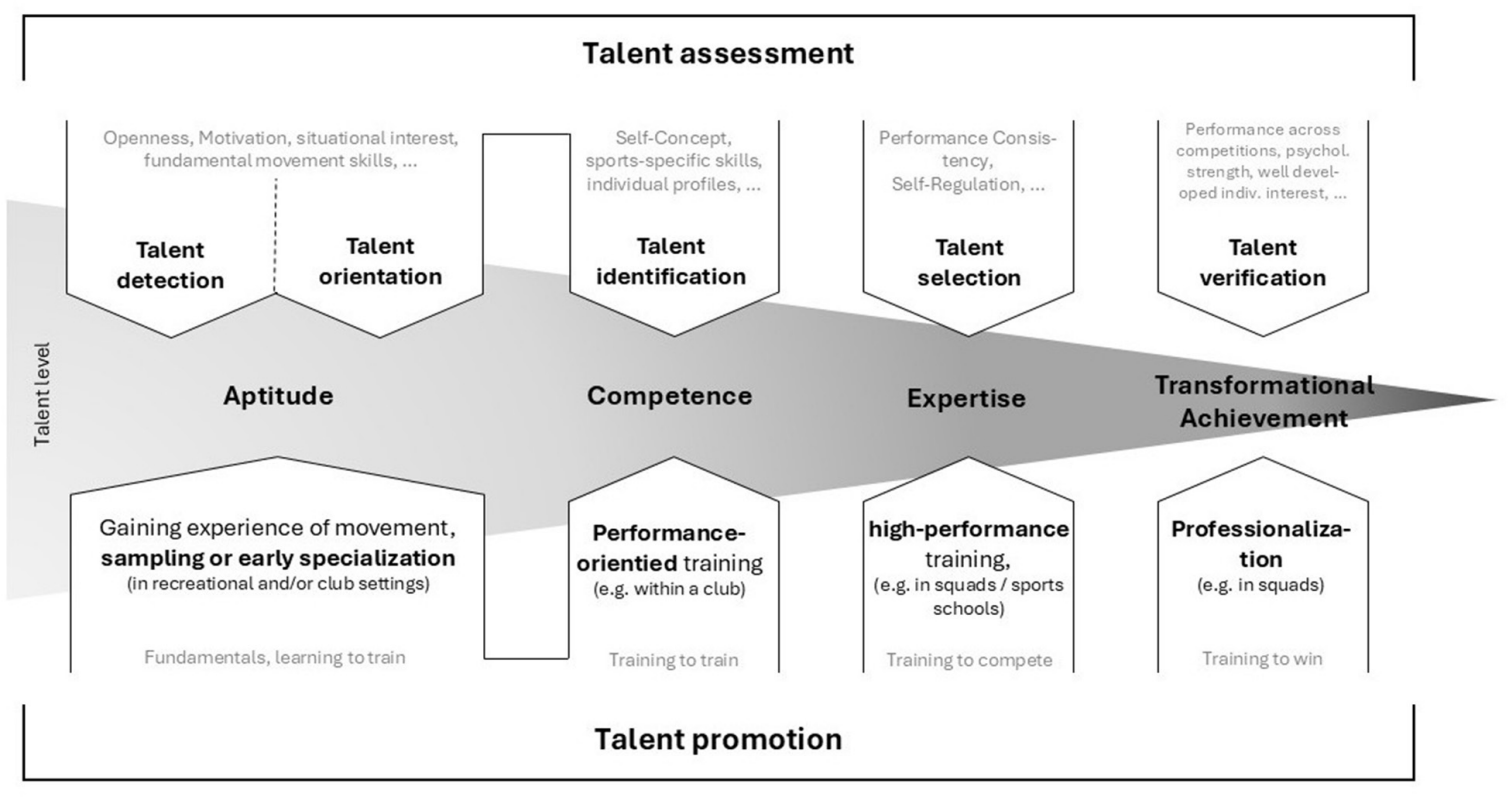
Integrative phase-based framework of Talent Assessment and Promotion (TAP) across the four phases: aptitude, competence, expertise and transformational achievement (based on Balyi and Hamilton, 2004; Hidi and Renninger, 2006; Hohmann, 2001; Hohmann and Seidel, 2003; Preckel et al., 2020).

Figure 1 illustrates the complete integrative framework. The vertical dimension represents the four developmental levels, while the horizontal dimension depicts the parallel perspectives of assessment and promotion. The figure explicitly highlights the non-disjunct nature of the levels and the soft transitions between them, emphasizing that developmental pathways are characterized by overlap, individual variability, and flexible timing rather than fixed stage boundaries.

### 3.1 Aptitude

The level of Aptitude represents the earliest segment within the integrative phase-based framework for Talent Assessment and Promotion (TAP). It integrates processes commonly described as talent detection and talent orientation, which, although closely related, serve distinct functions. Talent detection refers to the identification of general developmental potential within a broad and heterogeneous population. Talent orientation, in contrast, focuses on guiding individuals toward suitable sports, learning contexts, or developmental pathways based on their emerging profiles (Pion, 2024).

From an *assessment perspective*, the aptitude level emphasizes general, cross-sport characteristics that are not yet tied to specific sport contexts. Typical assessment content includes basic motor coordination, balance, speed, strength endurance, and fundamental movement skills (source). In childhood, these characteristics are often assessed using standardized motor test batteries (e.g. “Körperkoordinationstest für Kinder (KTK)”, Kiphard and Schilling, 2017) as part of regional talent assessment programs, which are designed to provide an initial overview of movement competence rather than to rank individuals for selection decisions (Höner et al., 2020; Vandorpe et al., 2012). In addition, predictors such as situational interest (Hidi and Renninger, 2006) or openness can be considered (Preckel et al., 2020, see Figure 1).

From a *promotion perspective*, the aptitude level is characterized by broad exposure to physical activity, typically low specialization, and diverse movement experiences. Promotion structures aim to facilitate access to organized sport participation and to create learning environments that support exploration across different sports and movement contexts (Fundamentals, Learning to Train; Balyi and Hamilton, 2004). Depending on the complexity of the sport, earlier and more rapid specialization may also be appropriate (Charbonnet and Conzelmann, 2024; Subotnik et al., 2011). However, assessment information primarily serves to inform orientation decisions and access to further developmental opportunities, rather than to promote early specialization or exclusion (Hohmann, 2009; Vaeyens et al., 2009).

Conceptually, the aptitude level corresponds to early phases in psychological models of talent development that emphasize general prerequisites rather than domain-specific competence. In Preckel's framework (2020), aptitude reflects broad abilities that may support later domain-specific development but are not yet associated with stable performance within a particular domain. Within the integrative framework, aptitude thus serves a development-opening function by structuring access to subsequent learning environments rather than anticipating later elite performance.

A central *challenge* at this level concerns the interpretation of assessment outcomes. Observed performance is strongly influenced by interindividual differences in biological maturation and prior movement experience, which can lead to pronounced performance differences that do not necessarily reflect underlying developmental potential (Malina, 2015). Empirical evidence regarding the relevance of aptitude-level characteristics beyond the transition to competence remains limited, underscoring the exploratory and provisional nature of assessment at this phase.

### 3.2 Competence

The level of Competence marks the transition toward increasing sport-specific differentiation within the TAP process. In this phase, individuals are no longer primarily explored for general potential but are evaluated within a specific sport context under increasingly representative training and performance conditions. Developmental pathways begin to stabilize (e.g., within a sports club with regular training sessions), while remaining open to further adaptation and refinement. Some children still have a second sport at this point (usually with lower priority).

From an *assessment perspective*, the competence level is characterized by a stronger focus on sport-specific technical, tactical, and physical characteristics. The aim is to identify talents for a specific sport (talent identification). In team sports, assessment commonly includes game-related performance indicators, tactical decision-making, and position-specific demands, whereas in individual sports, discipline-specific performance parameters such as times, distances, or technique quality become more salient (Höner and Feichtinger, 2016; Huijgen et al., 2013). In addition, psychological characteristics related to learning and performance regulation, such as self-regulation and persistence, are frequently considered, as they support adaptation to increasing training and competition demands (MacNamara et al., 2010; Toering et al., 2009). Importantly, even at the competence level, predictive validity remains phase-specific: assessment information is more informative for progression within the current developmental pathway than for forecasting long-term elite performance.

From a *promotion perspective*, the competence level coincides with the establishment of more structured and sport-specific development environments. Training frequency and intensity typically increase, competition becomes more formalized, and access to qualified coaching and support services expands. Within Long-Term Athlete Development models, this phase aligns with phases emphasizing systematic skill acquisition and consolidation, while within the FTEM framework it corresponds to entry into explicit talent development contexts (Training to train) (Balyi and Hamilton, 2004; Gulbin et al., 2013).

A key *challenge* at this level concerns the interpretation of assessment results. The specific requirement profiles of individual sports are difficult to delineate clearly, not only because of the wide range of different sports, but also due to substantial heterogeneity within sports, for example with regard to position- or role-specific demands (Döringer et al., 2025). Positions and roles within a sport often show considerable overlap with those in other sports, which complicates the clear identification of talent for one specific sport. At the same time, variance within athlete groups gradually decreases, increasing the risk that small performance differences are overinterpreted. Such differences may primarily reflect contextual influences, such as earlier learning opportunities or greater support, rather than stable developmental advantages (Johnston et al., 2018; Schorer et al., 2017).

### 3.3 Expertise

The level of Expertise reflects the consolidation of advanced sport-specific performance and follows the establishment of stable competence. Athletes it this phase demonstrate high levels of technical, tactical, and physical capability and are able to reproduce performance under demanding competitive conditions. Developmental pathways become increasingly shaped by performance requirements, and opportunities for alternative trajectories are reduced. At this point, athletes have specialized in one sport at the latest.

From an *assessment perspective*, expertise is characterized by a focus on performance robustness, adaptability, and consistency under competitive pressure. Typical assessment content includes sustained high-level performance across competitions, efficient skill execution in variable contexts, and psychological characteristics such as commitment, coping skills, and self-regulation (MacNamara et al., 2010; Sarkar and Fletcher, 2014). In this phase, the focus shifts toward distinguishing, within a given sport, those athletes who demonstrate the strongest prerequisites for elite performance (talent selection). In many sports, assessment relies predominantly on competition-based performance data collected at national or international levels.

From a *promotion perspective*, the expertise level is associated with highly structured and specialized development environments. Promotion strategies emphasize performance optimization, individualized training design, and careful management of training and competition load (Training to compete, Balyi and Hamilton, 2004). Within practice-oriented models, this phase corresponds to advanced talent development or early elite preparation. In Long-Term Athlete Development frameworks, it aligns with performance-oriented training phases, while within the FTEM framework it reflects advanced talent and early elite levels (Balyi and Hamilton, 2004; Gulbin et al., 2013). Importantly, expertise should not be equated with definitive elite success, as performance remains sensitive to contextual and developmental influences (e.g., training environments, access to facilities, financial support). Assessment and promotion are closely intertwined, as performance outcomes directly influence continued access to elite development environments and intensified support structures. This selection phase typically extends over several years. On this basis, selection decisions (such as squad membership) are made repeatedly and may be revised within the same phase. Simultaneously, other athletes may be selected or re-enter the pathway, resulting in a dynamic and cyclical selection process that varies across sports.

A central *challenge* at this level arises from the shrinking sample size. As athletes progress through earlier phases, group sizes decrease, limiting variability and increasing the consequences of diagnostic and promotion-related decisions. Longitudinal research shows that even at advanced phases, predictive validity remains constrained and non-linear developmental trajectories remain common (Güllich and Emrich, 2014). In addition, depending on the sport, athletes in this phase are faced with a fundamental decision as to whether they continue to invest heavily in their sport and subordinate other life domains to this pursuit, or whether they pursue a long-term career outside of sport. This decision is also strongly influenced by the availability and quality of support and promotion opportunities within the respective sport.

### 3.4 Transformational achievement

The level of Transformational Achievement represents the final segment within the integrative framework and corresponds to the phase of talent verification. In contrast to earlier levels, this phase is no longer concerned with identifying or selecting developmental potential, but with confirming and sustaining performance capability under the highest sport-specific demands. It typically coincides with transitions into senior elite competition, international performance contexts, or sustained participation at the highest national or professional levels.

From an *assessment perspective*, transformational achievement relies predominantly on direct performance outcomes in elite or near-elite competitive contexts. Diagnostic information is derived from sustained high-level performance across competitions and seasons, as well as from the ability to cope with intensified physical, psychological, and organizational demands. Compared with earlier levels, diagnostic uncertainty is reduced, as assessments are based on observable performance under highly representative conditions. In this phase, it can be assumed that athletes have developed a well-established and enduring form of domain-specific abilities and interest. In line with the four-phase model of interest development, this corresponds to a well-developed individual interest that is closely integrated into the athlete's personality and supports long-term engagement, self-regulation, and persistence at the elite level (Hidi and Renninger, 2006). Nevertheless, performance remains sensitive to contextual and environmental influences (e.g., coping with setbacks), which is why psychological strength is identified as a predictor at this level by Preckel et al. (2020).

From a *promotion perspective*, transformational achievement is embedded within highly individualized and specialized support structures. Promotion focuses on performance stabilization, load management, health protection, and long-term career sustainability (Quarrie et al., 2017). Within Long-Term Athlete Development frameworks, this level corresponds to phases emphasizing performance maintenance and optimization, while within the FTEM framework it aligns with elite and mastery-oriented levels (Balyi and Hamilton, 2004; Gulbin et al., 2013).

Despite its confirmatory nature, this level is not free from *challenges*. Successful verification reflects not only individual performance capability but also access to high-quality support systems and favorable environmental conditions. Organizational stability, coaching continuity, and broader life circumstances can substantially influence whether elite performance can be sustained over time. Consequently, non-verification in this phase does not necessarily indicate a lack of underlying competence but may instead reflect contextual constraints rather than individual limitations (injuries excluded).

## 4 Summary and outlook

The aim of the present paper was to contribute to conceptual clarity in Talent Identification and Development research by proposing an integrative, phase-based framework that structures the TAP process from talent detection to talent verification. Rather than introducing new predictors of future performance or replacing existing models, the framework provides a unifying structure that brings together established assessment-related, promotion-oriented, and practice-based approaches within a shared process logic (Pankhurst et al., 2013; Till and Baker, 2020).

A central contribution of the framework lies in its explicit clarification and structuring of process phases within TAP. Although previous research frequently refers to phases such as detection, orientation, identification, selection, or development, these terms have often been used inconsistently and without clear conceptual boundaries (Vaeyens et al., 2008). By defining and functionally distinguishing the phases of talent detection, talent orientation, talent identification, talent selection, and talent verification, the framework provides a common reference system that allows empirical findings, assessment practices, and promotion structures to be situated more precisely within the overall TID process. This clarification is particularly important given that the meaning of talent itself changes across phases, as different characteristics, performance expressions, and decision criteria become relevant at different levels of development (Preckel et al., 2020).

A further key contribution of the framework is the explicit conceptualization of assessment and promotion as parallel and continuously interacting dimensions across all phases of TAP. While the interdependence of assessment and promotion has long been acknowledged in sport science and developmental research, these perspectives are still often addressed separately, with assessment-focused studies emphasizing predictors and selection decisions and promotion-oriented models focusing on training structures and long-term pathways (Hohmann, 2009; Pankhurst et al., 2013). By explicitly linking both dimensions, the framework highlights that assessment outcomes are always embedded within specific promotion environments and that promotion structures, in turn, shape which performance characteristics become observable and diagnostically relevant. This perspective aligns with system-oriented and ecological approaches that conceptualize talent development as a dynamic and adaptive process rather than a linear sequence of decisions (Abbott et al., 2005; Baker et al., 2017; Zhao et al., 2024).

The framework is explicitly grounded in developmental theory by linking TAP phases to changing task demands and promotion-related levels. Developmental and interest-related models indicate that the relevance of talent-related characteristics systematically shifts over time, from broad, cross-domain prerequisites in early phases to increasingly sport-specific performance characteristics at later phases (Hidi and Renninger, 2006; Preckel et al., 2020). Embedding TAP phases within this developmental logic helps to explain why predictive validity is inherently phase-specific and limited in scope (Güllich, 2013; Johnston et al., 2018). Rather than representing a methodological deficit, limited long-term predictive validity can thus be understood as a structural feature of long-term developmental processes shaped by non-linearity, interaction effects, and contextual influences (Phillips et al., 2010).

At the same time, this perspective highlights several open questions that represent central challenges for future research. Despite a growing body of phase-specific evidence, systematic knowledge remains limited regarding how talent-related characteristics evolve across longer developmental time spans and across phase transitions. In particular, it is still unclear which characteristics retain relevance across multiple phases, which change their functional role over time, and which are informative only within specific developmental windows. Addressing these questions requires research designs that explicitly consider developmental timing, phase transitions, and the interaction between individual characteristics and promotion environments.

Beyond conceptual clarification, the proposed framework has important implications for research design and interpretation. By providing a phase-based structure, it enables researchers to specify more clearly which segment of the TAP process their findings address, thereby improving comparability across studies and reducing the risk of overgeneralization (Till and Baker, 2020).

From an applied perspective, the framework offers a conceptual tool for reflecting critically on the alignment between assessment practices and promotion structures within talent systems. Research on athletic talent development environments has shown that long-term success is shaped not only by individual characteristics but also by the quality, coherence, and adaptability of the surrounding system (Henriksen et al., 2010). By making explicit which assessment assumptions underlie specific phases and how these relate to promotion structures, the framework allows practitioners and organizations to examine whether selection criteria, training systems, and support structures are aligned with the developmental goals of each phase. In doing so, it highlights risks associated with premature specialization, early exclusion, and short-term performance orientation, which have repeatedly been shown to undermine long-term developmental efficiency (Güllich and Emrich, 2014).

Despite its contributions, the proposed framework also has limitations. As a conceptual framework, it does not provide operational definitions or measurement protocols for specific talent characteristics, nor does it prescribe optimal developmental pathways. Its primary value lies in structuring and integrating existing knowledge rather than in generating direct predictions of future performance. Future research is therefore needed to empirically examine how assessment criteria, promotion environments, and transition processes interact within and across phases, and how these dynamics vary across sports, performance levels, and sociocultural contexts.

In conclusion, this paper argues that progress in TAP research and practice depends less on identifying additional predictors of future success and more on achieving conceptual clarity regarding the structure and dynamics of the talent development process itself. By integrating assessment and promotion perspectives within a phase-based framework, the proposed framework provides a foundation for more coherent research, more transparent practice, and more meaningful interdisciplinary collaboration. Future research is invited to situate existing studies within this framework, to extend it where appropriate, or to engage in critical discussion. Clarifying TID in this way is not an endpoint, but a necessary step toward a more cumulative, developmentally informed, and context-sensitive science of talent in sport.
